# Melatonin protects ADSCs from ROS and enhances their therapeutic potency in a rat model of myocardial infarction

**DOI:** 10.1111/jcmm.12610

**Published:** 2015-06-17

**Authors:** Ping Zhu, Jianfeng Liu, Jinxin Shi, Qian Zhou, Jie Liu, Xianwei Zhang, Zhiyan Du, Qiaowei Liu, Yuanyuan Guo

**Affiliations:** a Department of Geriatric Cardiology, Chinese PLA General HospitalBeijing, China; b Shijingshan Teaching Hospital of Capital Medical University, Beijing Shijingshan HospitalBeijing, China; c Department of Cardiology, The Center Hospital of ZhoukouHenan Province, China; d The Health Department of Guard Bureau in the General StaffBeijing, China; e Institute of Basic Medical Sciences, Academy of Military Medical SciencesBeijing, China; f Beijing Institute of Radiation MedicineBeijing, China; g Department of Geriatrics, Civil Aviation General HospitalBeijing, China

**Keywords:** adipose tissue derived MSCs, melatonin, myocardial infarction, reactive oxygen species, rat model, apoptosis, viability, therapeutic strategy

## Abstract

Myocardial infarction (MI) is a major cause of death and disability worldwide. In the last decade, mesenchymal stem cells (MSCs) based cell therapy has emerged as a promising therapeutic strategy. Although great advance have been made using MSCs to treat MI, the low viability of transplanted MSCs severely limits the efficiency of MSCs therapy. Here, we show evidence that *ex vivo* pre-treatment with melatonin, an endogenous hormone with newly found anti-oxidative activity, could improve survival and function of adipose tissue derived MSCs (ADSCs) *in vitro* as well as *in vivo*. ADSCs with 5 μM melatonin pre-treatment for 24 hrs showed increased expression of the antioxidant enzyme catalase and Cu/Zn superoxide dismutase (SOD-1), as well as pro-angiogenic and mitogenic factors like insulin-like growth factor 1, basic fibroblast growth factor, hepatocyte growth factor (HGF), epidermal growth factor. Furthermore, melatonin pre-treatment protected MSCs from reactive oxygen species (ROS) induced apoptosis both directly by promoting anti-apoptosis kinases like p-Akt as well as blocking caspase cascade, and indirectly by restoring the ROS impaired cell adhesion. Using a rat model of MI, we found that melatonin pre-treatment enhanced the viability of engrafted ADSCs, and promoted their therapeutic potency. Hopefully, our results may shed light on the design of more effective therapeutic strategies treating MI by MSCs in clinic.

## Introduction

Myocardial infarction (MI), which is defined pathologically as myocardial cell death due to prolonged myocardial ischaemia, may lead to sudden death or severe haemodynamic deterioration 1. It is a major cause of death and disability worldwide. In the United States alone, MI affects a total of 7.6 million people, and every year the associated mortality exceeds 125,000 people 2. Despite the advance of therapeutic approaches, traditional therapies do not address the central problem, namely the loss of functional myocardium and the blockage of small blood vessels. In the last decade, mesenchymal stem cells (MSCs) based cell therapy has emerged as a promising therapeutic strategy for chronic and degenerative diseases because of their characteristics such as self-renewal, multidirectional differentiation, paracrine abilities, weak immunogenicity, easy isolation and expansion, and ethic advantage. Great advance have been gained using MSCs to treat MI, both in pre-clinical studies 3–6 and in clinical trials 7–10.

However, a severe challenge limits the efficiency of MSCs therapy for MI is the low viability of transplanted MSCs. It has been demonstrated that more than 80–90% of grafted cells died within 72 hrs 11. After transplantation, the engrafted MSCs are exposed to an extremely harsh, pro-apoptotic micro-environment in the infracted heart 12. The high concentration of reactive oxygen species (ROS), resulting from cell necrosis and inflammation 13, impairs MSCs function as well as triggers apoptosis 14. Therefore, it is of great interest to find a simple approach to protect transplanted MSCs from the crucial environment.

Melatonin, or *N*-acetyl-5-methoxytryptamine, which was first discovered in the pineal gland in the 1950s 15, is a widely distributed molecule involved in a variety of physiological processes, such as sleep promotion, immune defense and proliferation control 16,17. Recently, it has been reported that melatonin showed antioxidant and anti-inflammatory properties 18. Wang *et al*. reported protective effect of melatonin on bone marrow MSCs (BM-MSCs) against hydrogen peroxide induced apoptosis *in vitro* 19. In addition, Tang *et al*. reported that melatonin pre-treatment could improve the survival and function of transplanted MSCs after focal cerebral ischemia 20, while Mias *et al*. demonstrated that 21 *ex vivo* pre-treatment with melatonin could improve the survival, pro-angiogenic/mitogenic activity, and efficiency of MSCs injected into ischaemic kidney. However, whether melatonin pre-treatment can protect adipose tissue derived MSCs (ADSCs) against the harsh environment in the infracted heart, as well as the underlying mechanism, remain unknown.

In this present study, we examined the effect of melatonin treatment on rat ADSCs. *In vitro* studies showed that melatonin treatment increased the expression of antioxidant enzymes such as catalase and Cu/Zn superoxide dismutase (SOD-1), as well as mitogenic factors in MSCs. Furthermore, we found that melatonin significantly protected MSCs from H_2_O_2_ triggered apoptosis. The *in vivo* studies were performed using a rat model of MI. Bioluminescence imaging indicated that melatonin pre-treatment promoted the retention and survival of transplanted MSCs in the heart region. Notably, significantly enhanced therapeutic efficiency was achieved using MSCs pre-treated by melatonin.

## Materials and methods

All animal experiment procedures in this study were conducted in compliance with the Guide for the Care and Use of Laboratory Animals (NIH publicationno. 85-23, revised 1996) and were approved by the Institutional Animal Care and Use Committee of Chinese PLA General Hospital.

### ADSCs isolation and culture

Mesenchymal stem cells were isolated from the adipose tissues in the subcutaneous inguinal region of 8–10 weeks old male Sprague–Dawley (SD) rats (about 250 g), following protocol previously reported 22. In general, the adipose tissues were digested with 0.075% type I collagenase (Sigma-Aldrich, St Louis, MO, USA) in a 5% CO_2_ incubator at 37°C for 1 hr. The resulting mixture was filtered through a 140 μm nylon mesh, followed by centrifuged at 200 × g for 10 min. at 4°C. After washing, the pellet was loaded onto a Percoll density gradient (1.077 g/ml) and finally centrifuged at 100 × g for 20 min. at 4°C. The resulting interphase was washed and cultured in DMEM-low glucose (DMEM-LG; Gibco, Grand Island, NY, USA) supplemented with 10% foetal bovine serum (MDgenics, St. Louis, MO, USA) in a 5% CO_2_ incubator at 37°C. After four to five passages expansion, the MSCs were characterized by flow cytometry before transplantation.

### Flow cytometry

For flow cytometry analysis, MSCs were trypsined and washed with phosphate buffered solution (pH 7.4), followed by incubation in the dark for 1 hr at room temperature with CD90-FITC, CD29-FITC, CD45-FITC, CD34-FITC and CD31-FITC (BD Pharmingen, San Diego, CA, USA) antibodies. The fluorescence of 10,000 cells was analysed on FACScalibur (Becton Dickinson, Franklin Lakes, NJ, USA) using Cell Quest Pro software.

### Melatonin pre-treatment and Immunoblot analysis

Mesenchymal stem cells were treated with 5 μM melatonin or PBS as control for 24 hrs and extensively washed with PBS. The 24 hrs incubation time was selected following previous report showing that expression of the antioxidant enzyme catalase and the cytokine basic fibroblast growth factor (b-FGF) increased in a time-dependent manner after treatment with melatonin, reaching a maximum between 16 and 24 hrs 21. In some experiments, the non-selective melatonin receptor antagonist luzindole was added at 10 μM 1 hr prior to the addition of melatonin. H_2_O_2_ was used as an exogenous ROS source. The MSCs were treated by 20 μM H_2_O_2_ for 1 hr for the adhesion assay and western blot assay, or 24 hrs for the apoptosis assay.

For western blot analysis, MSCs were washed twice in cold PBS and lysed in buffer containing 20 mM Tris (pH 7.5), 150 mM NaCl, 1 mM Na_2_-EDTA, 1 mMEGTA, 1% Triton, 2.5 mM sodium pyrophosphate, 1 mM b-glycerophosphate, 1 mM Na_3_VO_4_, 1 mg/ml of leupeptin and 1 mM phenylmethylsulphonylfluoride. Protein concentrations were determined by Bradford Protein Assay Kit (Bio-Rad, Hercules, CA, USA). Proteins were separated in a 10% sodiumdodecyl sulphate–polyacrylamide gel and transferred to a poly-vinylidenedifluoride membrane (Chemicon International Inc., Temecula, CA, USA). After blocking with Tris-buffered saline (TBS) based 5% non-fat milk containing 0.1% Tween 20 for 1 hr at room temperature, the membrane was washed twice with TBS-T and incubated with primary antibody including anti-p Focal Adhesion Kinase (FAK), anti-p Src, anti-catalase, anti-SOD-1 and anti-cleaved caspase-3, respectively, overnight at 4°C. After washing, the membrane was incubated for 1 hr at room temperature with horseradish peroxidase-conjugated secondary antibodies. Bands were detected by enhanced chemiluminescence reagent (Santa Cruz Biotechnology, Santa Cruz, CA, USA). Quantifications of band intensities were made using the Photo-Image System (GE Life Sciences, Pittsburgh, PA, USA). Actin was used as internal control.

### Adhesion assays

Mesenchymal stem cells with different treatment were suspended to a concentration of 3 × 10^4^ cells and plated to each well of 48-well plates with or without the presents of 20 μM H_2_O_2_. These cells were allowed to attach for 1 hr at 37°C in 5% CO_2_. Then plates were carefully washed three times with PBS, and four random fields were photographed with a phase-contrast microscope. The adhered cells were counted blinded. Each experiment was performed in triplicate wells and repeated at least three times.

### Dihydroethidium staining

Dihydroethidium (DHE) staining was used to investigate the intra-cellular level of ROS in H_2_O_2_ treated MSCs following a modified protocol that reported by Cai *et al*. 23. In brief, the similar amounts of MSCs with or without melatonin treatment were seeded onto glass cover slips in six-well plates. After adhesion, these cells were cultured for an additional 6 hrs with 20 μM H_2_O_2_. Dihydroethidium was diluted in PBS to a concentration of 5 μM, and 200 μl DHE–PBS solutions were dropped onto the cell monolayer and incubate at 37°C in dark for 30 min. Then, the excess DHE was rinsed off with PBS twice, and the cover slips were mounted to microscopic slides using mounting media. The cells were examined under fluorescence microscopes (Olympus, Tokyo, Japan) at excitation and emission wave-lengths of 520 and 610 nm, respectively. The intensity of DHE staining was quantified using IPWIN60 software (Media Cybernetics, Inc., Rockville, MD, USA) expressed as the ratio of 4′,6-diamidino-2-phenylindole (DAPI) staining. The untreated cells stained with DHE were used as control and set as 100%.

### RT-PCR analysis

The expression levels of VEGF, insulin-like growth factor 1 (IGF-1), b-FGF, HGF, epidermal growth factor (EGF) and granulocyte-colony stimulating factor (G-CSF) in melatonin or PBS treated MSCs were analysed by RT-PCR. Total RNA was extracted with Trizol reagent (Invitrogen, Carlsbad, CA, USA). 2 μg RNA were reverse-transcribed into cDNA by random primers using Technical Bulletin Reverse Transcription System (Promega, Madison, WI, USA). Real-time PCR analysis was performed on Corbett6200 using SYBR Green PCR mix (TOYOBO Co., Osaka, Japan). Ct values of GAPDH (as internal control) were subtracted from Ct values of the genes of interest (ΔCt). ΔCt values of genes from MSCs treated with PBS were set as 100%. The primers for VEGF, IGF-1, b-FGF, HGF, EGF and G-CSF were purchased from Qiagen (Quanti-Tect Primer Assays, Qiagen Inc., Germantown, MD, USA). The Primers for GAPDH were synthesized by Invitrogen using the following sequence (Sense; Antisense): 5′-CTCCCAACGTGTCTGTTGTG-3′ and 5′-TGAGCTTGACAAAGTGGTCG-3′.

### Apoptosis assay

The MSCs with or without melatonin pre-treatment were treated with 50 μM H_2_O_2_ for 24 hrs. These cells were stained using AnexinV apoptosis kit (Sigma-Aldrich) and examined under fluorescence microscopes (Olympus). Then, these MSCs were trypsined and analysed on FACScalibur (Becton Dickinson) using Cell Quest Pro software.

### Rat MI model

Myocardial infarction model rats were established following protocol previously reported 24. Briefly, 8–10 week-old SD male rats were anaesthetized with sodium pentobarbital (40 mg/kg). The hearts were exposed by left side limited thoracotomy. Then, the left anterior descending coronary artery was ligated 3 mm below its origin using a 6-0 silksuture. Ischaemia was confirmed by the blanching of the myocardium and dyskinesis of the ischaemic region, while restoration of normal rubor indicated successful reperfusion. These MI model rats were randomly assigned into four groups for MSCs transplantation.

### Labelling of MSCs and transplantation

Mesenchymal stem cells were labelled with luciferase (Fluc) and green fluorescent protein (GFP) using a commercially available lentivirally transduction kit (Cyagen Co., Ltd., Guangzhou, China). The efficiency of transduction and labelling were confirmed under fluorescence microscopes (Olympus) and FACScalibur (Becton Dickinson).

For transplantation, after surgical occlusion of the left anterior descending coronary artery, 2 × 10^6^ MSCs (with or without melatonin pre-treatment) in 100 μl PBS, or 100 μl PBS with/without melatonin were injected intramyocardially at three sites in the border of the infarct area. The four groups of MI rats with transplantation were as follows: Group 1 (*n* = 8): PBS injection, as control; Group 2 (*n* = 8): Transplantation with MSCs pre-treated by PBS; Group 3 (*n* = 8): Transplantation with MSCs pre-treated by melatonin; and Group 4 (*n* = 8): injection of melatonin in PBS (5 μM).

### Bioluminescence imaging

Bioluminescence imaging was performed using a Xenogen IVIS 100 imaging system (Caliper Life Sciences, Hopkinton, MA, USA) with bioluminescence charge-coupled device camera.

For *ex vivo* imaging, MSCs of different numbers, 1, 2.5, 5, 7.5, 10 and 12.5 × 10^4^ per-well, were seeded onto 96 well plate in 100 μl DMEM-LG medium with 10% FBS, respectively. Thereafter, 100 μl D-luciferin (Caliper, Hopkinton, MA, USA) solution (30 μg/ml) were added at room temperature. The MSCs were then analysed under the camera using the Living Image 4.0 software (Xenogen).

For *in vivo* imaging, rats were tracked on days 1 and day 7 after transplantation. Being anaesthetized with sodium pentobarbital (40 mg/kg), the rats were injected intraperitoneally with 100 mg/kg D-luciferin (Caliper). The animals were placed in the imaging chamber and BLI signals were detected by creation of polygonal regions of interest over the pericardium. Signals were acquired for 5 min. at a 2-min. interval until peak signal was observed. Peak signals were estimated using the Living Image 4.0 software (Xenogen).

### Assessments of LV function

4 weeks after MSCs transplantation, the LV functions of MI model rats were evaluated by echocardiography examination. After being anaesthetized with sodium pentobarbital (40 mg/kg), the rats were examined for echocardiography by Sequoia512 Color Ultrasound System (Siemens, Munich, Germany). The LV end-systolic diameter (ESD), LV end-diastolic diameter (EDD) and LV ejection fraction (LVEF) were measured.

### Analysis of heart histology

4 weeks after MSCs transplantation, rats from all four groups were sacrificed. The hearts were removed and embedded in paraffin. Sections were made longitudinally from the mid-left ventricle and base. All sections were stained with Masson’s Trichrome. The infarcted size and wall thickness of each image were measured using the Image Pro Plus software. All histological data in this study were treated and analysed by three persons blinded.

In addition, immunohistochemistry was performed to measure capillary density in the infarcted myocardium. The paraffin sections was stained with rabbit polyclonal anti-von Willebrand antigen (Abcam, Inc., Cambridge, MA, USA ). For quantification, capillaries were counted in five randomly chosen fields at 20× and the mean number of capillaries per field was used for statistical analysis.

### Statistical analysis

All quantified data represent an average of at least triplicate samples. The error bars represent the standard deviation of the mean. Differences between groups were calculated for significance by one-way anova with a Tukey test using GraphPad Prism software version 5 (Graph-Pad Software, LaJolla, CA, USA). A *P* < 0.05 was considered significant.

## Results

### Characterization of ADSCs

**Figure 1:**
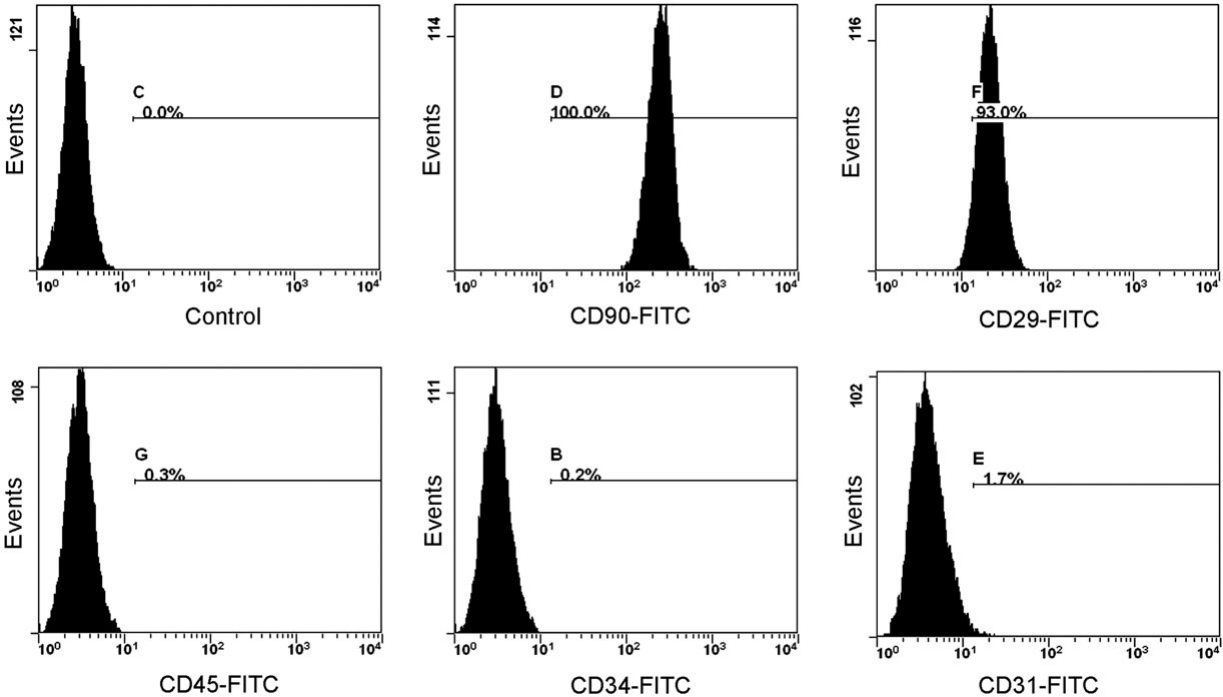
Characterization of ADSCs. Flow cytometry analysis of cultured ADSCs. These MSCs were positive for mesenchymal stem cells markers CD90, CD29 and negative for haematopoietic markers CD45, CD35 and CD31.

Mesenchymal stem cells were isolated from the adipose tissues of SD rats by standard procedures. These ADSCs, which adhered to plastic and showed a fibroblastic appearance, were further characterized using flow cytometry for their expression of MSCs markers. As shown in Figure1, most of the MSCs we isolated were positive for CD90 (100%) and CD29 expression (>93%), and negative for haematopoietic markers CD34 (0.2%), CD31 (1.7%) and CD45 (0.3%).

### Melatonin enhances the antioxidative property of MSCs

**Figure 2:**
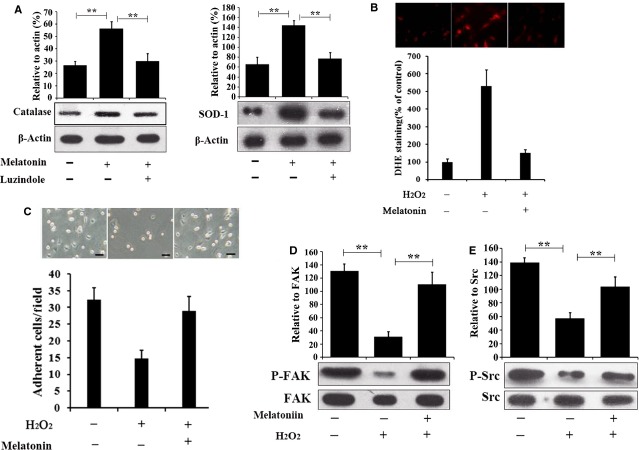
Melatonin enhances the anti-oxidative property of MSCs. (A) Expression of anti-oxidant enzymes catalase and SOD-1 of MSCs with different treatment were examined by western blot. The MSCs were treated with PBS, 5 μM melatonin or 5 μM melatonin together with the nonselective melatonin receptor antagonist luzindole (10 μM, added 1 hr prior) for 24 hrs. Actin was used as internal control. (B) DHE stain for intra-cellular level of ROS in PBS or melatonin pretreated MSCs exposed to 20 μM H_2_O_2_ for 6 hrs. Representative images and statistics were shown. The DHE staining intensity of untreated cells was used as control and set as 100%. (C) Adhesion assay of MSCs with or without melatonin pretreatment exposed to 20 μM H_2_O_2_ for 1 hr. Represented image and statistics were shown. (D and E) Phosphorylation of FAK and Src were analyzed by western blot in MSCs with indicated treatment. The phosphorylation level of untreated MSCs was set as 100%. **P < 0.01 compared between groups as indicated.

As reported, the ROS environment in the infarcted heart is a major obstacle to transplanted MSCs’ function and viability. To test whether melatonin pre-treatment enhanced the antioxidative property of MSCs, we first examined the effect of melatonin on MSCs’ expression of antioxidant enzymes such as catalase and Cu/Zn SOD-1. As seen in Figure2A, western blot analysis showed an induced expression of both catalase and SOD-1 by MSCs after treatment with 5 μM melatonin for 24 hrs, whereas co-treatment with the melatonin receptor antagonist luzidole blocked this induction. Next, the MSCs, pre-treated by melatonin or PBS as the control, were challenged exogenously with H_2_O_2_. The intro-cellular ROS level of MSCs was analysed by DHE staining. As shown in Figure2B, the ROS level in MSCs that pre-treated with melatonin was significantly decreased. Also, the adhesion assay was performed as ROS has been reported to impair MSCs’ adhesion. As shown in Figure2C, H_2_O_2_ significantly hindered the adhesion of MSCs, whereas pre-treatment with melatonin rescued this impairment. Furthermore, the phosphorylation of adhesion associated kinases FAK and Src were examined by western blot assays, as they play crucial roles in cellular focal adhesion by regulating the actin cytoskeleton. Compared with PBS treated MSCs that exposed to H_2_O_2_, the expression of p-FAK and p-Src were significantly revived in melatonin pre-treated MSCs, as shown in Figure2D and E. Together, these data indicate that melatonin pre-treatment could enhance the antioxidative property of MSCs.

### Melatonin protects MSCs from apoptosis induced by exogenous ROS

**Figure 3:**
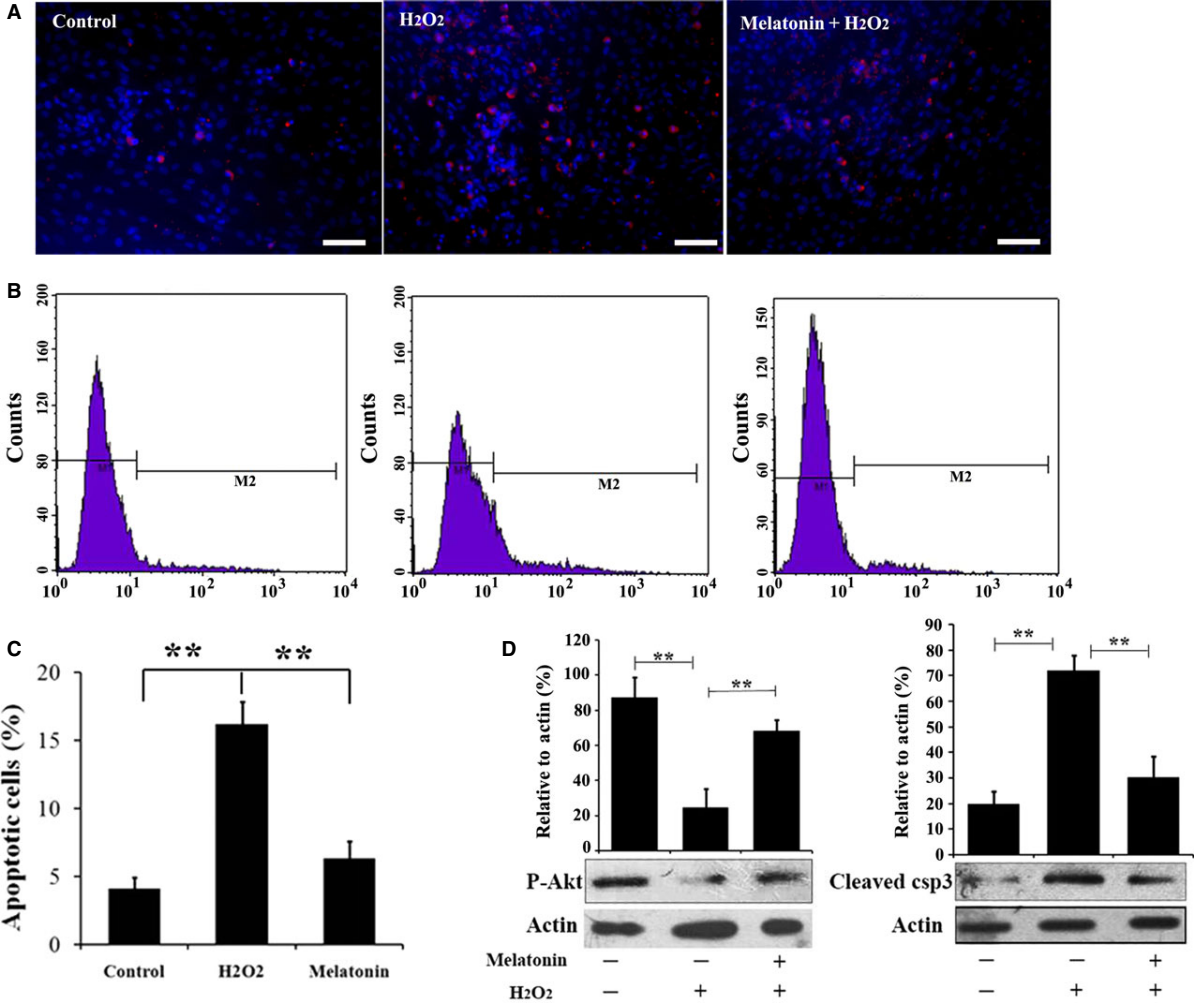
Melatonin protects MSCs from apoptosis induced by exogenous ROS. (A–C) Apoptosis assay of MSCs with or without melatonin pretreatment exposed to 50 μM H_2_O_2_ for 24 hrs. Fluorescence microscopy (A) and flow cytometry analysis (B and C) were performed to detect cell apoptosis using Anexin V apoptosis kit. **P < 0.01. (D) Phosphorylation of Akt and level of cleaved caspase 3 in MSCs with indicated treatment were examined by western blot. The level of p-Akt and cleaved caspase 3 in untreated MSCs were set as 100%, respectively, **P < 0.01.

We next examined the effect of melatonin on exogenous ROS induced apoptosis of MSCs. As shown in Figure3A–C, H_2_O_2_ triggered a significant increase in apoptosis level in PBS treated MSCs, while MSCs with melatonin pre-treatment shared a similar apoptotic rate with control MSCs. Furthermore, western blot assays for the survival associated kinase p-Akt and apoptosis associated protein cleaved caspase-3 confirmed this observation (Fig.3D). All together, the above data suggest that pre-treatment with melatonin could protect MSCs from exogenous oxidative stress and increase MSCs survival *in vitro*.

### Melatonin stimulates the secretion of proangiogenic and mitogenic factors by MSCs

**Figure 4:**
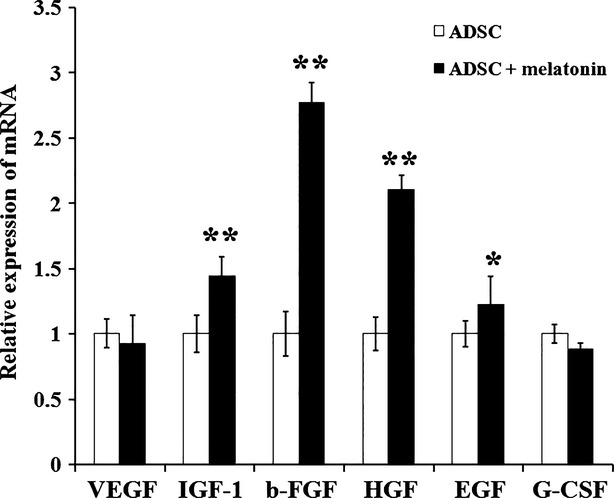
The effect of melatonin pretreatment on MSCs expression of paracrine factors. The relative expression level of several pro-angiogenic and mitogenic factors in melatonin (5 μM; 6 hrs) pretreated MSCs were examined by quantitative RT-PCR. The expression level of each factor in untreated MSCs was set as 100%. *P < 0.05, **P < 0.01.

The paracrine of proangiogenic and mitogenic factors by MSCs play important roles in MSCs based therapy for MI. Therefore, we examined the mRNA levels of VEGF, IGF-1, b-FGF, HGF, EGF and G-CSF in MSCs with or without melatonin treatment. Notably, after 6 hrs treatment with 5 μM melatonin, the relative expression of IGF-1, b-FGF, HGF, EGF were significantly up-regulated, whereas the expression of VEGF and G-CSF were unmodified (Fig.4). These data suggest that melatonin could also support MSCs’ function by stimulating their paracrine of proangiogenic and mitogenic factors.

### Melatonin pre-treatment promotes the retention and survival of transplanted MSCs in a MI rat model

**Figure 5:**
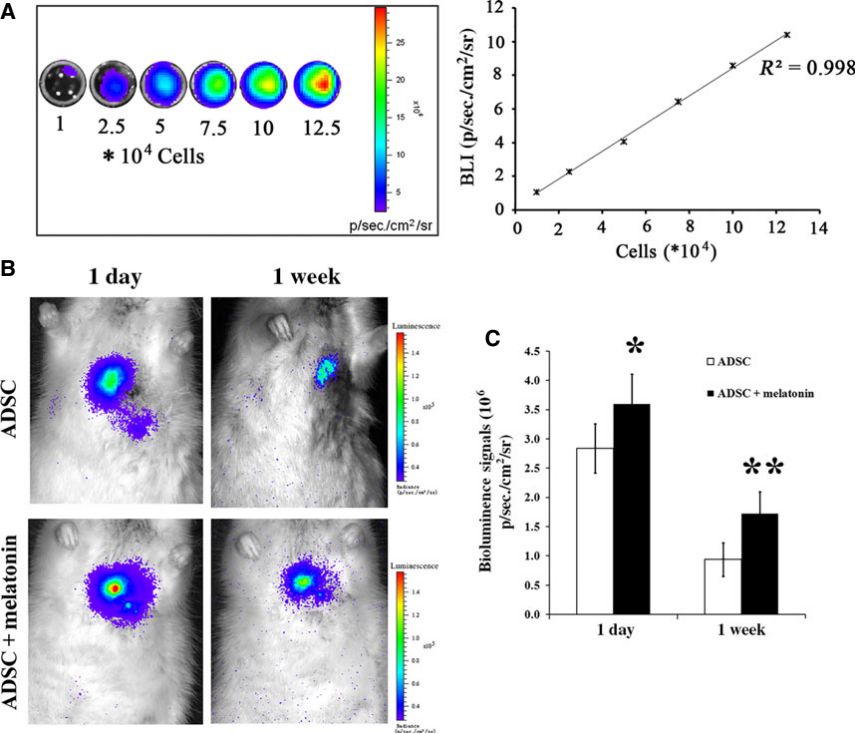
Melatonin pretreatment promotes the retention and survival of transplanted MSCs in rat MI model. (A) *Ex vivo* bioluminescence imaging shows a linear relationship between cell number and Fluc reporter gene. (B) *In vivo* bioluminescence imaging of MSCs with or without melatonin pretreatment detected at days 1 and 7 after transplantation. (C) Quantification of BLI signals. *P < 0.05, **P < 0.01.

To examine the effect of melatonin pre-treatment on MSCs survival *in vivo*, the MSCs were lentivirally transduced to express firefly luciferase (Fluc) as well as GFP before transplantation into the infract region of MI model rats. As shown in Figure5A, *Ex vivo* bioluminescence imaging demonstrated that the number of MSCs was linearly correlated with BLI signals (*R*^2^ = 0.998), suggesting that the reporter could be used to quantitatively track MSCs. The same amounts of MSCs with or without melatonin pre-treatment were injected, and rats receiving transplantation were imaged longitudinally 1 and 7 days after transplantation. As shown in Figure5B and C, the survival rate of both melatonin pre-treated and untreated MSCs decreased as days passed by, with remaining MSCs in day 7 only a half number of those in day 1. However, the intensity of bioluminescence signal in melatonin pre-treated MSCs group is significantly higher than that in PBS pre-treated MSCs group at each time point, especially at day 7, where the signal of melatonin pre-treated MSCs group was twofold higher. These results indicate that the melatonin could promote the retention and survival of transplanted MSCs *in vivo*.

### Melatonin pre-treatment enhanced MSCs therapeutic potency in the MI rat model

**Figure 6:**
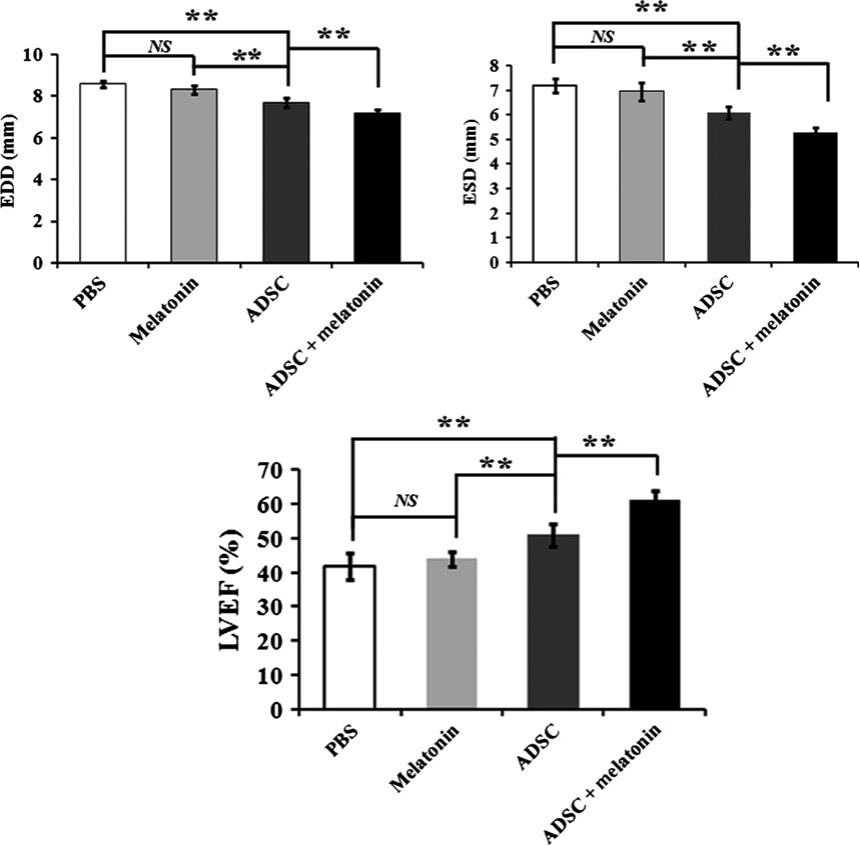
Assessment of LV functions of MI model rats after MSCs transplantation. Four weeks after transplantation of MSCs with or without melatonin pretreatment (PBS and melatonin injection as control), the LV functioning parameters including the LV ejection fraction (LVEF) and LV fractional shortening (LVFS) were examined by echocardiography. *NS*: no significance, **P < 0.01.

Four weeks after transplantation, we assessed the LV parameters using echocardiography examination. As shown in Figure6, parameters including the LVEF and the LV ESD and EDD indicated a significant amelioration after transplantation with MSCs. Notably, a significantly enhanced therapeutic efficiency was observed in the group transplanted with melatonin treated MSCs, compared with that with untreated MSCs. Injection of melatonin in PBS demonstrated some improvement on LV parameters, but no significant difference was observed compared with PBS control.

**Figure 7:**
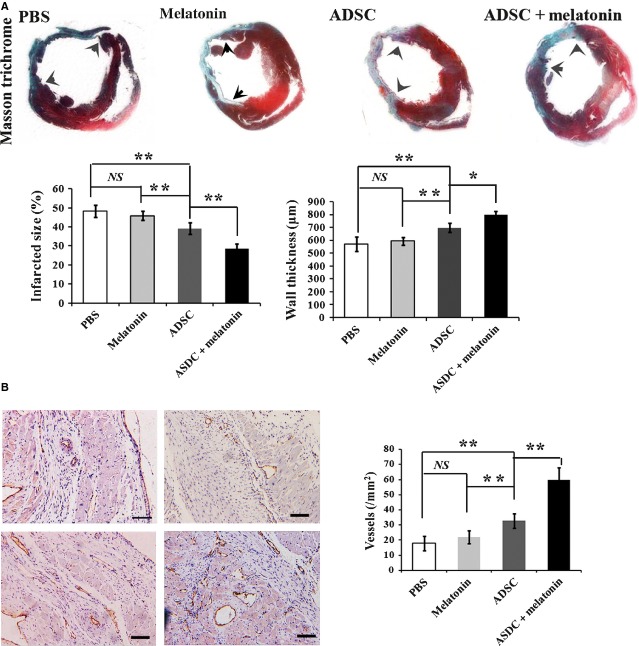
Heart histology of MI model rats after MSCs transplantation. (A) Four weeks after transplantation by MSCs with or without melatonin pre-treatment, the hearts of MI model rats were sectioned along long axis and stained with Masson’s Trichrome. The infarcted size and heart wall thickness were measured. Representative images and quantification of infarcted size and heart wall thickness were shown. *N* = 8 per group. *NS*: no significance, *P < 0.05, **P < 0.01. Arrows indicating infarction area. (B) Capillary density in the infarcted myocardium was analyzed by immunohistochemistry. von willebrand antigen (VWAg) was used as marker for blood vessels. Representative images and quantification of vessel density were shown. *NS*: no significance, **P < 0.01.

In addition, at the end of week 4, the MI model rats were sacrificed and the hearts were sectioned longitudinally. The infarct size and wall thickness of hearts were measured after Masson’s Trichrome staining. Compared with PBS control group, transplantation with either melatonin treated or untreated MSCs reduced the infarct size and increased the wall thickness, while melatonin treated MSCs showed an elevated efficiency (Fig.7A). No significant difference was observed either between PBS and melatonin injection group. Furthermore, capillary density in the infarcted myocardium was measured by immunohistochemistry. The mean micro-vessel count per field was significantly higher in the group transplanted with melatonin pre-treated MSCs than that with untreated MSCs (Fig.7B). Taken together, these data suggest that melatonin pre-treatment could enhance MSCs’ therapeutic efficiency in the MI rat model.

## Discussion

In the last decade, MSCs based cell therapy for MI and other degenerative diseases has achieved great advance and been considered a promising strategy 25–27. However, the low retention and survival of engrafted MSCs remains a major challenge. In our current study, we show that melatonin pre-treatment could up-regulate the expression of antioxidative enzymes as well as mitogenic factors in MSCs, protecting MSCs from H_2_O_2_ induced adhesion impairment and apoptosis *in vitro*. Using a rat model of MI, we find that MSCs with melatonin pre-treatment have enhanced viability in the MI region, and significantly reduced the infarct size, increased the wall thickness, and elevated capillary density in the infarcted myocardium, resulting in a promotion of the heart function. To our knowledge, this is the first evidence that melatonin, the natural hormone without major side effects, could protect ADSCs from ROS and enhance their therapeutic potency for MI.

In the study, rat models of acute MI were used, that is, cell transplantation was performed immediately after coronary artery ligation. It was noteworthy that the therapeutic mechanism of acute MI with MSCs transplantation was different from that of established heart failure. As for acute MI, the main role of cell transplantation would be the prevention of LV remodelling. After coronary artery ligation, or coronary artery occlusion in clinic, cardiac cells in and surrounding the ischaemic region would be impaired and die gradually, leading to the progressive remodelling and functional deterioration of heart 28. When stem cells were transplanted immediately or early after coronary artery ligation, they could play important roles in preventing the pathological progression of LV remodelling *via* paracrine effects (inhibiting apoptosis, necrosis and fibrosis, promoting revascularization). This has been confirmed to be a main mechanism through which stem cells repair acute MI 29,30. However, the case is different in established heart failure (2 weeks after MI). As lots of cardiac cells have been lost, improvement of the pathological condition in established heart failure would be mainly depended on the cardiac regeneration 26. Therefore, in established heart failure, transplantation of engineered cardiac tissues should be a more effective option 31–33.

Accumulating studies are now focusing on elevating the survival and function of MSCs after engrafting. For example, it has been reported that hypoxia pre-conditioning could enhance the therapeutic effect of MSCs 34,35. Also, pre-treating MSCs with, or combining MSCs treatments with growth factors such as VEGF 36,37, FGF-2 38 and tumour necrosis factor-α 38, have been shown to promote MSCs adhesion, survival and therapeutic potency in MI. Furthermore, several groups modified MSCs genetically to induce the expression of anti-apoptotic proteins, such as Akt 39, Bcl-2 40, or growth factors such as SDF-1 41, VEGF 42 and HGF 43. However, since the complexity of the micro-environment, increased concentration of certain growth factors may cause unexpected side effect. In addition, safety issues must be considered when introducing genetic modified stem cells into human body.

Melatonin (*N*-acetyl-5-methoxytryptamine) is an endogenous hormone secreted from the pineal gland and a variety of other tissues 44. Besides regulating various physiological processes, such as circadian rhythm, sleep and reproduction, melatonin has been reported to reduce tissue injury by decreasing oxidative damage, modulate immune response and stimulate cytokine secretion in BM cells. Recently, protective effect of melatonin on BM-MSCs has been reported *in vitro* 19, as well as *in vivo* when treating focal cerebral ischaemia 20, ischaemic kidney 21, acute lung ischaemia–reperfusion injury 45 and other disease models such as acute interstitial cystitis 46. Although the organs and tissues were variant in the above studies, melatonin protected engrafted MSCs from the intensively oxidative and inflammatory micro-environment resulting from the ischaemia. In consistent, we here show that melatonin pre-treatment could protect ADSCs from ROS and promote their therapeutic efficiency treating MI. Because melatonin is currently used as a dietary complement in human with no major side effects, pre-treating MSCs with melatonin might be a promising strategy to enhance MSCs viability and functions in clinic trials, as little safety issues of melatonin should be of concern. Of note, melatonin itself has been reported to show protective effect against isoproterenol bitartrate-induced myocardial injury in rat 47. Also, in the above mentioned studies 45,46, injection of melatonin alone was compared with applying melatonin and MSCs systemically, where melatonin itself did show certain therapeutic potency. In our study, melatonin alone was also injected intramyocardially, but no significant improvement on heart function was observed. We supposed that this may be due to the limited dose and rapid loss of melatonin through intramyocardial injection. Continuous infusion through caudal vein may be more effective, which deserved further investigation in future.

When MSCs are transplanted into the infarcted heart, they face with an extremely harsh micro-environment. The necrosis of cells and the inflammatory response may increased the concentration of ROS over threefold in the MI region 13. Melatonin pre-treatment activated the expression of anti-oxidant enzymes such as catalase and SOD-1 in MSCs, resulting in maintaining a relatively low intra-cellular ROS level when the environment ROS is high, as shown in Figure2A and B. This up-regulation of antioxidant enzymes seems to be dependent on melatonin receptors on MSCs, because the melatonin receptor antagonist luzindole reversed this effect. Besides causing apoptosis directly, ROS could also impair the adhesion ability of MSCs, leading to a further increase of apoptosis due to inadequate interactions between cells and the matrix, termed anoikis 24. Interestingly, we found that melatonin pre-treatment restored the adhesion ability and the p-FAK and p-Src level in MSCs exposing to H_2_O_2_. Moreover, the autocrine and paracrine of angiogenic and mitogenic factors are key mechanisms of MSCs function in treating MI. IGF-1, HGF and EGF are important mitogenic factors promoting proliferation of multiple types of cells, and b-FGF could contribute to angiogenesis. Notably, we found that melatonin pre-treatment induced the expression of these growth factors. However, we did not observe the up-regulation of VEGF and G-CSF. This may be caused by a different mechanism of melatonin signalling in MSCs, which awaits further exploration. In principle, our present study showed that melatonin treatment protect MSCs from ROS and enhance their function by: (*i*) induction of antioxidant enzymes; (*ii*) Restoration of adhesion and (*iii*) Promotion of growth factors expression. However, the mechanism of melatonin’s protection on MSCs still remains unclear. Our further study will focus on the signalling pathways, as well as the effect of melatonin on the other factors impairing MSCs survival and function in the MI environment, such as iNOS, immune cells and cytokines.

Conventionally, BM-MSCs were widely used in MSCs based cell therapies, because they were the first discovered adult stem cells and relatively well-studied. Recently, ADSCs have gained much attention and been used as an alternative because liposuction is less invasive than BM aspiration and can be easily and reproducibly replicated in a large-scale. Although there are differences between BM-MSCs and ADSCs, such as several different cell surface markers and different tendency of differentiation, no significant difference in their mechanisms and efficiency treating MI has been reported 48. Up to now, BM-MSCs have been used in most of the studies focusing on the strategy to enhance MSCs survival in treating MI, but the evidence on ADSCs was lacking, in spite of the promising future of ADSCs in cell therapy. Thus, our results, showing that ADSCs could also be protected by melatonin pre-treatment and lead to a significant amelioration of MI, may be helpful in clinic.

Several different methods can be used to track engrafted MSCs and determine their viability. For example, Tang *et al*. reported the use of Y chromosome-specific quantitative RT-PCR to examine the survival rate of implanted sex-mismatched allogeneic stem cell transplantation in the recipient heart 49. Also, the MSCs could be pre-labelled with fluorescent markers such as GFP, DAPI, Dil, ect., and cryo-sections of heart are made and examined under fluorescence microscopy. In this study, we chose to label MSCs by lentivirally transduction of vectors expressing luciferase and GFP, and bioluminescence imaging was used to determine the viability of MSCs. Unlike the other methods, the bioluminescence imaging is direct-viewing, and the animals need not to be killed on different time points. Of note, in this present study, we chose day 7 as a time-point in which bioluminescence imaging was performed to verify the survival rate of engrafted MSCs. The bioluminescence imaging data from the 14th day post-transplantation or later time-points would be useful to further validate the conclusion. In fact, we have also tried to obtain these data in imaging experiments. However, as is known, the *in vivo* bioluminescence signals could be detected only when the number of survived cells reach a certain threshold. For cells engrafted in rat heart, the threshold is relative high, as the signals need to pass through the sternum, thick muscle and skin. Due to the loss of the survived cells in hearts with transplant time, detectable bioluminescence signals attenuated with time. At 14th day post-transplantation and later time-point, we hardly detected visible signals in most animals, including control MSC group and melatonin-pre-treated MSC group. Meanwhile, the expression level of transfected luciferase gene also attributed to this issue. Although we obtained stable transfected MSCs by lentivirally transduction, higher luciferase expression was of demand for bioluminescence imaging. Better transduction approaches and vectors are being tested in our ongoing further studies, to improve the luciferase expression in MSCs for better tracking.

In conclusion, our data showed that melatonin pre-treatment for 24 hrs up-regulated ADSCs’ expression of the antioxidant enzymes such as catalase and SOD-1, and protected MSCs from ROS induced apoptosis both directly by promoting anti-apoptosis kinases like p-Akt as well as blocking caspase cascade, and indirectly by restoring the ROS impaired cell adhesion. In addition, the expression proangiogenic and mitogenic factors like IGF-1, HGF, EGF and b-FGF were also elevated. Using a rat model of MI, we found that melatonin pre-treatment enhanced the viability of engrafted ADSCs, and promoted their therapeutic potency. Hopefully, our results may shed light on the design of more effective therapeutic strategies in clinic treating MI and other diseases by MSCs.

## Funding

This work was partly supported by grants from the National Natural Science Foundation of China (8135002), the Clinical Support Foundation of Chinese PLA General Hospital (2012FC-TSYS-2004, 2013FC-TSYS-2015) and the Central Health Special Research Projects (13BJZ26).

## Conflicts of interest

None declared.
